# Dispersal influences genetic and acoustic spatial structure for both males and females in a tropical songbird

**DOI:** 10.1002/ece3.3456

**Published:** 2017-10-24

**Authors:** Brendan A. Graham, Daniel D. Heath, Daniel J. Mennill

**Affiliations:** ^1^ Department of Biological Sciences University of Windsor Windsor ON Canada; ^2^ Great Lakes Institute of Environmental Research University of Windsor Windsor ON Canada

**Keywords:** natal dispersal, sex‐biased dispersal, song learning, songbird, spatial genetic structure

## Abstract

Animals exhibit diverse dispersal strategies, including sex‐biased dispersal, a phenomenon common in vertebrates. Dispersal influences the genetic structure of populations as well as geographic variation in phenotypic traits. Patterns of spatial genetic structure and geographic variation may vary between the sexes whenever males and females exhibit different dispersal behaviors. Here, we examine dispersal, spatial genetic structure, and spatial acoustic structure in Rufous‐and‐white Wrens, a year‐round resident tropical bird. Both sexes sing in this species, allowing us to compare acoustic variation between males and females and examine the relationship between dispersal and song sharing for both sexes. Using a long‐term dataset collected over an 11‐year period, we used banding data and molecular genetic analyses to quantify natal and breeding dispersal distance in Rufous‐and‐white Wrens. We quantified song sharing and examined whether sharing varied with dispersal distance, for both males and females. Observational data and molecular genetic analyses indicate that dispersal is female‐biased. Females dispersed farther from natal territories than males, and more often between breeding territories than males. Furthermore, females showed no significant spatial genetic structure, consistent with expectations, whereas males showed significant spatial genetic structure. Overall, natal dispersal appears to have more influence than breeding dispersal on spatial genetic structure and spatial acoustic structure, given that the majority of breeding dispersal events resulted in individuals moving only short distances. Song sharing between pairs of same‐sex animals decreases with the distance between their territories for both males and females, although males exhibited significantly greater song sharing than females. Lastly, we measured the relationship between natal dispersal distance and song sharing. We found that sons shared fewer songs with their fathers the farther they dispersed from their natal territories, but that song sharing between daughters and mothers was not significantly correlated with natal dispersal distance. Our results reveal cultural differences between the sexes, suggesting a relationship between culture and sex‐biased dispersal.

## 1 INTRODUCTION

Animals exhibit diverse dispersal strategies that influence their ecology and evolution (Clobert, Le Galliard, Cote, Meylan, & Massot, 2009). Dispersal strategies vary both among and within species and often show pronounced differences between the sexes (Greenwood, 1980; Greenwood & Harvey, 1982). Sex‐biased dispersal is common in birds and mammals; females usually disperse farther than males in birds, whereas the reverse is true for mammals (Clarke, Sæther, & Roskaft, 1997; Greenwood, 1980; Wolff, 1994). Dispersal is a critical component of the ecology, evolution, and spatial distribution of all animals and has profound effects on the genetic and phenotypic structure of populations (Bohonak, 1999; Clobert et al., 2009; Ellers & Slabbekoorn, 2003; Tarwater & Beissinger, 2012).

Behavioral traits, such as acoustic signals, play a critical role in mate attraction and territory defense (Bradbury & Vehrencamp, 2011). Whereas most animals develop vocalizations without the influence of vocal learning, a restricted group of animals learn their vocalizations by listening to conspecifics, including humans, some birds, bats, elephants, seals, and cetaceans (Janik & Slater, 1997; Jarvis, 2004; Poole, Tyack, Stoeger‐Horwath, & Watwood, 2005; Sanvito, Galimberti, & Miller, 2007). In birds, vocal learning is common to three groups: songbirds, parrots, and hummingbirds (Jarvis, 2004). By studying geographic variation in learned vocalizations in relation to dispersal patterns, we have a unique opportunity to examine how animal movement shapes acoustic variation (Salinas‐Melgoza & Wright, 2012; Wright & Wilkinson, 2001). Many animals learn their vocalizations early in life, and animals dispersing long distances may introduce new songs from their natal neighborhoods into their breeding neighborhoods (Lynch, 1996). In this case, patterns of spatial genetic structure should correspond with patterns of spatial acoustic structure (i.e., population‐wide patterns of song sharing). However, the new songs that move with immigrants into a breeding population will only become established if other birds learn those songs (Payne, 1996). If there is selection for animals to sing local songs, dispersal may have little influence on the acoustic structure of a population (Beecher & Brenowitz, 2005). In this case, patterns of genetic structure and patterns of acoustic structure may be markedly different.

Population variation has been well‐studied in songs produced by male birds (Podos & Warren, 2007), but not female birds. Female song is uncommon in North Temperate ecosystems (but see Garamszegi et al., 2007), but it is widespread in the tropics (Slater & Mann, 2004). Female song is understood to be the ancestral trait in Oscine birds (Odom, Hall, Riebel, Omland, & Langmore, 2014). Systems where both sexes sing are ideal for between‐sex vocal comparisons, especially for learned traits like bird song, because dispersal to novel environments can affect the transmission and hence variation of these signals (Pavlova et al., 2013). Current models examining the relationship between dispersal and acoustic variation have focused solely on male birds (Ellers & Slabbekoorn, 2003). Given that female songbirds often disperse further from natal territories than males do, they may exhibit different spatial patterns of acoustic variation from male songbirds (Mennill & Rogers, 2006). Therefore, between‐sex comparisons offer a compelling system to examine the role of dispersal on acoustic variation because of the prevalence of sex‐biased dispersal in birds.

In this study, we examine dispersal, spatial genetic structure, and spatial acoustic structure in male and female Rufous‐and‐white Wrens (*Thryophilus rufalbus*), resident songbirds found in Central America and northern South America. In this species, both sexes possess song repertoires (males: 11.4 ± 0.3, range = 8–15; females: 8.5 ± 0.7, range = 4–11), although males have significantly larger repertoires than females (Harris, Wilson, Graham, & Mennill, 2016; Mennill & Vehrencamp, 2005). Males and females use the same vocal repertoire to produce solo songs or songs that are part of coordinated vocal duets (Mennill & Vehrencamp, 2005). Some song types are sex‐specific, whereas other songs types are shared between males and females (Mennill & Vehrencamp, 2005). Even though there is the potential for individuals to learn songs from the opposite sex (as observed in other species, Evans & Kleindorfer, 2016), measurements of song sharing and acoustic similarity suggest that males learn primarily from other males, and that females learn from other females, as suggested in other species (Mennill & Rogers, 2006). Juvenile Rufous‐and‐white Wrens appear to continue to learn songs following natal dispersal, further allowing us to study the role between dispersal and song variation (Graham, 2016).

To study the interplay between dispersal and acoustic variation, we sought to answer three questions in this study. (1) Is dispersal sex‐biased in this species? To answer this question, we quantify both natal dispersal distance (i.e., the movement of young animals from their natal territory to their first breeding territory) and breeding dispersal distance (i.e., the movement of an adult animal from one breeding territory into another within or between years) in Rufous‐and‐white Wrens. Additionally, we examined spatial genetic structure to determine whether genetic data support re‐sight/recapture observations. (2) Does natal dispersal or breeding dispersal shape genetic and acoustic spatial structure? To answer this question, we compare natal dispersal distances with breeding dispersal distances to quantify and contrast juvenile dispersal and adult dispersal. (3) Finally, is there a relationship between dispersal and acoustic variation? Current models of song learning have emphasized the role that both intersexual selection and dispersal play on acoustic divergence (Ellers & Slabbekoorn, 2003). We attempt to extend these models by considering the influence of dispersal on acoustic variation for both sexes. Given the prevalence of sex‐biased dispersal in birds (Clarke et al., 1997; Greenwood, 1980), our study system offers a compelling opportunity to explore the role of dispersal on acoustic variation, allowing us to extend upon current models that have focused on this relationship exclusively in male birds.

To answer these three questions, we analyzed two different data sets, and made predictions about the relationship between dispersal and genetic structure and acoustic structure. In the first data set, we examine spatial genetic structure and spatial acoustic structure (i.e., population‐wide patterns of song sharing) for both sexes. If animals show little or no dispersal, then we predicted individuals would be more closely related to neighbors than non‐neighbors, and that individuals would exhibit greater rates of song sharing with neighbors than non‐neighbors. If, however, one or both sexes disperse greater distances, then we predicted individuals would exhibit limited or no spatial genetic structure and limited or no spatial acoustic structure. In the second data set, we analyze rates of song sharing between sons and fathers, and mothers and daughters. Similar to our previous predictions, if animals disperse only short distances, then we predicted we would observe a strong correlation between distance and the rate of song sharing between parents and offspring because those that remain close to their parents will continue to learn from their parents. In contrast, if one or both sexes disperse greater distances, and assuming song learning continues following dispersal, then we predicted we would observe no relationship between song sharing and dispersal from natal territories.

## 2 METHODS

**Figure 1:**
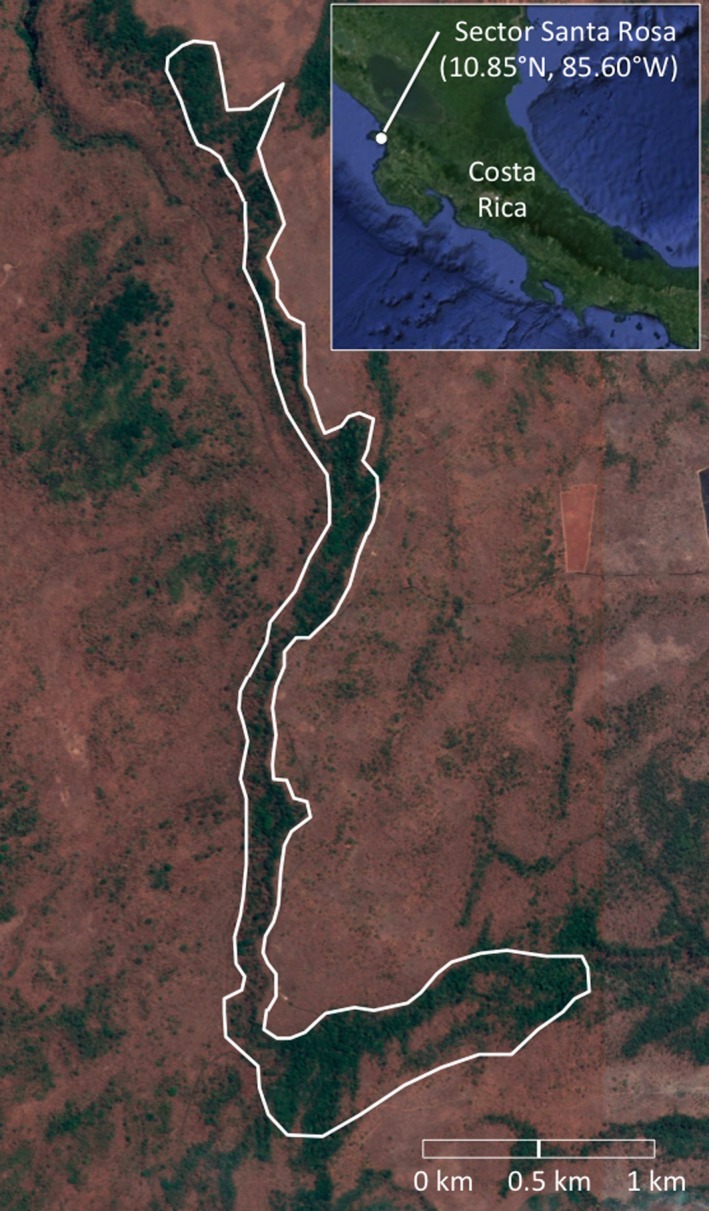
Map of study area, showing the distribution of the breeding areas of Rufous‐and‐white Wrens in sector Santa Rosa of the Area de Conservación Guanacaste. Base map images from Google Maps

From 2003 to 2013, we monitored a population of Rufous‐and white Wrens in Sector Santa Rosa of the Guanacaste Conservation Area in northwestern Costa Rica (10°51′N, 85°36′W; 286 m a.s.l.). We captured birds using mist nets and banded each animal with a unique band combination consisting of one numbered aluminum band and three color bands. We collected a small sample of blood (~100 μl) from the brachial vein and stored blood samples in 95% ethanol or Queen's Lysis Buffer (Seutin, White, & Boag, 1991). Rufous‐and‐white Wrens are sexually monochromatic; we determined the sex of individuals based on the presence of a brood patch (females) and by singing behavior (sexes can be distinguished based on fine‐structural differences in songs; Mennill & Vehrencamp, 2005). Each year we identified all the birds in our study site, which is a 7‐km‐long patch of mature evergreen Neotropical dry forest surrounded by less‐mature seasonal Neotropical dry forest (Figure 1). Annually, we collected data on birds’ territory locations, breeding partners, and breeding activities. In addition to banding adult birds, we also banded nestlings. We banded nestlings when they were 7–12 days old, collecting small blood samples and providing each nestling with one numbered aluminum band and one color band.

### 2.1 Estimating dispersal

We measured the natal dispersal distance and breeding dispersal distance of both male and female Rufous‐and‐white Wrens. We define “natal dispersal” as the movement from an individual's natal territory to their first breeding territory. We define “breeding dispersal” as the movement of a breeding adult from an established breeding territory to another breeding territory (Greenwood, 1980; Yáber & Rabenold, 2002). We considered the first breeding territory to be the territory where we observed an animal during its first breeding year.

Over the 11 years of this study, we banded 230 nestlings, and we used recapture/re‐sight data to identify natal dispersal events. In total, we re‐sighted 21 individuals (9.1% of all banded juveniles). Nest depredation rates are extremely high in our population (up to 90%; Topp & Mennill, 2008), as is common in tropical ecosystems (Martin, 2015); the low percentage of recaptured birds likely reflects high rates of predation. In addition to the dispersal events we observed, other banded birds may disperse outside of the boundaries of our study population further contributing to our low recapture rates; genetic analysis of among‐population structure has detected gene flow between Santa Rosa and nearby populations (Graham, 2016), and therefore, some of the banded nestlings may have dispersed outside of the study population.

Additional pairs of parent–offspring dyads may exist in our study population, even when the offspring were not banded. This could occur for several reasons. (1) In some cases, nests were too high for us to reach, or nests were placed on trees growing in inaccessible terrain, over rivers or cliffs. Nestlings were not banded in these cases. (2) Due to a long breeding season, some nestlings may have hatched after we had left our field site; we banded nestlings in June and July of each year, but birds may have bred until the end of August (Stiles & Skutch, 1989). At the beginning of each breeding season, approximately one‐third of the adult birds were unbanded, and some of these new birds may have come from these two situations. Therefore, we used genetic analysis to identify parent–offspring dyads, and increase our pool of natal dispersers. We used 10 variable DNA microsatellites (see below) to identify potential parent–offspring dyads.

To quantify breeding dispersal, we used recapture/re‐sight data of banded adults. Over the 11 years of our study, we banded 237 adult birds (134 males and 103 females). We measured breeding dispersal distance following the same approach as our estimates of natal dispersal. We considered a breeding dispersal event to have occurred when an individual was found in an alternate territory (in most cases with a different mate), either within the same breeding season or between consecutive breeding seasons.

We quantified natal dispersal and breeding dispersal using two different measurements. First, we calculated straight‐line distances between the center of a bird's natal territory and first breeding territory using the geographic distance calculator in GenAlEx 6.5 (Peakall & Smouse, 2006, 2012). Second, we measured dispersal distance as the number of breeding territories that an individual dispersed across (Cockburn, Osmond, Mulder, Green, & Double, 2003; Sankamethawee, Hardesty, & Gale, 2010).

We compared differences in natal and breeding dispersal distances between sexes using generalized linear models (GLMM) with a negative binomial error structure. We constructed six separate models; two each for natal dispersal and breeding dispersal, and two to compare natal and breeding dispersal. For our models comparing natal dispersal or breeding dispersal between sexes, we either used dispersal distance or the number of territories dispersed as our dependent variables and sex as our independent variable. For the two models comparing natal and breeding dispersal, again we used dispersal distance or the number of territories dispersed as our dependent variables and dispersal type (i.e., natal or breeding) as our dependent variable. Additionally, we were interested in determining if adult males or adult females were more likely to exhibit breeding dispersal. For this analysis, we compared the number of male dispersers and non‐dispersers to the number of female dispersers and non‐dispersers using a Fisher's exact test.

### 2.2 Genetic analyses

We extracted DNA from blood samples using a Wizard Extraction Kit (Promega) and genotyped 213 individuals (123 males and 90 females) at 10 microsatellite loci. We genotyped all individuals using four existing microsatellite primer sets *ThPl 14*,* ThPl 20*,* ThPl 30* (Brar et al., 2007), *RWWR 2c* (H. Mays, personal communication), and six new microsatellite primer sets (*Tru 08*,* Tru 11*,* Tru 18*,* Tru 20*,* Tru 24*,* Tru 25*). We developed the new microsatellites primer sets using a modified method of the Fischer and Bachman (1998) microsatellite enrichment procedure (Walter, Ovenden, & Heath, 2007). All PCRs were conducted in 12.5 μl reactions with 1 μl of genomic DNA. PCR cocktails contained 1.25 μl of 10× PCR buffer (Applied Biosystems), 0.5 μl of MgCl_2_ (2.5 mmol/L), 0.45 μl of dNTPs (0.2 mmol/L), 0.05 μl of bovine serum albumin, and 0.5 U of Taq (Genscript, Applied Biosystems). For the primer sets *Tru 08*,* Tru 11*,* Tru 18*,* Tru 20*,* Tru 24*,* Tru 25*, and *RWWR 2c*, PCR cocktails included 1 μmol/L each of an M13 tailed‐forward primer, reverse primer, and a 5′ IR‐dye‐labeled M13 primer (GTAAAACGACGGCCAGT). PCR cocktails for primer sets *ThPl 14*,* ThPl 20*, and *ThPl 30* contained 1 μmol/L each of the forward primer and the IR‐dye‐labeled reverse primer and used the same PCR amplification profiles described in Douglas, Heath, and Mennill (2012). The remaining primer sets used the following amplification profiles one cycle of 94.0°C for 2 min, followed by 34 cycles of 94.0°C for 10 s, 50.0°C for 10 s, 72.0°C for 30 s, followed by a final extension cycle of 72.0°C for 90 s; for primer set *Tru* 24, we increased the annealing temperature (*T*_2_) to 54.0°C to reduce stutter. PCR products were visualized using a LiCor 4300 DNA analyzer (LiCor Biosciences, Inc.), and allele sizes were scored using GeneImagIR 4.05 (Scanalytics, Inc., Rockville, MD). Finally, we included known size standards on each run to ensure that all gels were scored and sized consistently across all gels.

The ten loci used in the analysis were polymorphic, ranging from low to high variability (mean allelic richness = 7.57 ± 1.29). Mean observed heterozygosity was 0.59 ± 0.08, while the mean expected heterozygosity was 0.66 ± 0.09 across all 10 loci. Two loci (*ThPl 14* and *ThPl 30*) showed significant deviations from Hardy–Weinberg equilibrium (*p* < .001), and two of 45 pairwise locus combinations showed evidence of linkage disequilibrium following corrections for multiple comparisons (*p* < .001). Deviations from Hardy–Weinberg equilibrium could be indicative of null alleles; however, we used all 10 loci and accounted for potential null alleles in our analysis (see below).

We calculated relatedness between individuals using software ML‐Relate (Kalinowski, Wagner, & Taper, 2006). ML‐Relate uses a maximum‐likelihood approach to estimate the probability that two individuals share an allele identical by descent at a given locus. Unlike other available software, ML‐Relate can compensate for the presence of null alleles (Kalinowski et al., 2006), giving a more accurate estimate of relatedness between individuals. The program classifies individuals into four different relationship categories: parent–offspring, full‐siblings, half‐siblings, and unrelated. Given the goals of our study, we focused exclusively on identifying parent–offspring relationships. Null alleles can pose a problem in parentage analysis and potentially result in false parentage exclusions (Dakin & Avise, 2004), and therefore, we tested for heterozygosity deficiency using the Monte Carlo randomization test available in ML‐Relate (Guo & Thompson, 1992). The program identified three loci (*Tru 08*,* ThPl 14*, and *ThPl 30*) with high probabilities of heterozygote excess (*p* < .001), so we specified these three loci as having null alleles for our analysis. When null alleles are specified, the program estimates the frequency of null alleles following the methods of Kalinowski and Taper (2006).

To validate all parent–offspring dyads identified using ML‐Relate, we used the specific “hypothesis testing” function in ML‐Relate. This function tests the probability of a putative relationship (i.e., parent–offspring) versus an alternative relationship (i.e., unrelated, half‐sibling, or full‐sibling). For this analysis, we compared all parent–offspring relationships against full‐sibling relationships, given that full‐sibling relationships are most likely to be misidentified as parent–offspring relationships (Woltmann, Sherry, & Kreiser, 2012). We tested all putative parent–offspring relationships by simulating 10,000 genotypes and only rejected the alternative hypothesis (full‐sibling) if *p* < .05. In all instances, we identified the putative parent (i.e., father or mother) and offspring (i.e., son or daughter) using our banding data. We considered the bird that was banded at the earlier date to be the parent, and the bird that was banded at the later date to be the offspring (e.g., 2007 vs. 2008). Additionally, we incorporated breeding data to help us correctly identify true parent–offspring relationships. For example, if the program failed to reject the alternative hypothesis (full‐sibling) for two males, we compared the putative offspring's genotype to the putative father's female partner from the previous breeding season. If a bird did not match for both parents, we considered this to be a Type I error (i.e., individuals that are not related but shared alleles across all loci by chance; Christie, 2010). The rate of extra‐pair copulations and paternity is low in this species (2% of all nestlings and 6% of all nests; Douglas et al., 2012), so it seems unlikely that a high proportion of mismatches with putative fathers would be due to extra‐pair paternity. Furthermore, ML‐Relate correctly rejected the null hypothesis (that animals were full‐siblings and not parent‐offspring) for all of the known 13 parent–offspring dyads identified by re‐sight/recapture data that were included in our genetic analysis (genotyping data were not available for offspring or parents for 8 of the 21 natal dispersal events identified with recapture/re‐sight data), thereby demonstrating the effectiveness of this method to correctly identify parent–offspring dyads in our dataset.

### 2.3 Song analysis

We recorded the songs of individuals during the breeding season, in April through July of each year of the study, a time of year when vocal output is high for this species (Topp & Mennill, 2008). We recorded each individual on at least two separate occasions. The majority of our recordings (60%) were collected during focal recordings: We followed each bird around its territory (each morning, for 1–2 hr between 04:45 and 11:00 hr) and confirmed the bird's band combination during the recording. We recorded songs during focal recordings using a solid‐state digital recorder (Marantz PMD‐660 or PMD‐661; 44.1 kHz sampling rate; 16‐bit accuracy; WAVE format) and a shotgun microphone (Sennheiser MKH70 or ME67/K6). We supplemented focal recordings with recordings from automated recorders (see Harris et al., 2016 for details). We placed these recorders within the center of the territories of each focal pair, often within 10 m of the focal pair's nest. We confirmed that the songs collected by these automated recorders were those of the intended pair by re‐sighting the focal individuals in their territory after automated recording sessions and by matching the songs collected by the automated recorders to the songs collected during focal recordings (as in Harris et al., 2016).

### 2.4 Song‐type assignment and song sharing

We annotated all audio files using SYRINX‐PC sound analysis software (J. Burt, Seattle, Washington, USA), and we built a library of all the song types in the repertoire of each male and female. To classify song types, we inspected the fine‐structural characteristics of songs following the approach outlined in Harris et al. (2016). Previous work by Barker (2008) has shown that discriminant analysis can differentiate song types based on fine‐structural measurements (i.e., duration of song, maximum frequency, minimum frequency, and intersyllable interval). Based on these findings, we considered songs to be different when (1) they sang different sequences or frequencies of introductory syllables, (2) they sang trills composed of different elements, or produced at different frequencies (>100 Hz difference) or delivered at different rate (trills were considered different if they were delivered at a rate >2 syllables/s), and (3) they sang terminal syllables that had a different shape on the sound spectrogram (Graham, 2016).

For our analysis of song sharing, we focused exclusively on song sharing within each sex. Although males and females share some song types, sharing between sexes is low, suggesting that young males learn primarily from other males, while young females learn from other females (Mennill & Vehrencamp, 2005). To measure song sharing, we calculated an adjusted Jaccard's coefficient of sharing, *S*_j_, using the following formula (Tracy & Baker, 1999),

$$S_{j}=c/\left(\left(a+b+c\right)-d\right)$$

where *a *= the number of song types in individual A's repertoire but not individual B's, *b *= the number of song types in individual B's repertoire but not individual A's, *c *= the number of song types shared between the two individuals, and *d *= the difference in repertoire size between individual A and B. We chose this coefficient because it accounts for differences in repertoire size (*d*) and birds in our population showed considerable variation in repertoire size (Harris et al., 2016).

### 2.5 Spatial genetic structure analysis

To examine patterns of fine‐scale genetic structure and determine if Rufous‐and‐white Wrens exhibit sex‐biased dispersal, we used spatial autocorrelation analysis (Smouse & Peakall, 1999). Spatial autocorrelation measures how closely correlated a variable is across geographic space. Previous work has shown that spatial autocorrelation is robust and capable of detecting patterns of sex‐biased dispersal even when there are subtle differences in dispersal between sexes (Banks & Peakall, 2012). Unlike other spatial analyses (e.g., Mantel tests) where raw geographic distances are compared, spatial autocorrelation separates distances into classes. We used 1 km as our minimum geographic distance class for this analysis. We chose this value based on the distribution of individuals throughout our study site; the farthest gap between established territories in our study site is 1 km, and we feel that this is a biologically relevant distance for our species. This value is similar to distances used in other spatial genetic studies of nonmigratory bird populations (e.g., Liebgold, Gerlach, & Ketterson, 2013). Distance classes were combined into four separate distance classes for our analysis (1, 2, 3, and 6 km). We combined all of the farthest distances into a single distance class 6 km, following the approach of Liebgold et al. (2013), because we had fewer samples at >3 km, and combining them together gave us a larger sample size that was comparative to the sample sizes for our closest three distance classes.

For each distance class, GenAlEx calculates a coefficient of correlation (*r*), ranging between −1 and 1, to measure how similar, dissimilar, or random the genetic relationship among individuals is within distance classes. A significant positive value of *r* indicates that individuals are more genetically similar than is expected by chance, while a negative significant *r* indicates that individuals are less closely related than is expected by chance. When the value of *r* is not significantly different from zero, this indicates random spatial distribution, where individuals are just as likely to be situated next to closely related individuals as they are to unrelated individuals. In addition to calculating *r*, spatial autocorrelation in GenAlEx uses bootstrapping methods to generate upper and lower 95% confidence intervals around *r* (Peakall, Ruibal, & Lindenmayer, 2003).

We compared overall patterns of spatial genetic structure and patterns of spatial genetic structure between sexes using the “multiple population analysis” in GenAlEx. This analysis combines datasets from multiple populations (in this case, males and females) to produce a single correlogram that depicts the common spatial pattern across all populations. We generated separate genetic and geographic pairwise matrices for each sex; we used straight‐line distance (km) between individuals as our measurement of geographic distance, and Nei's genetic distance as our measurement of genetic distance. We chose to analyze together all individuals genotyped across the 11 years (123 males and 90 females), rather than comparing patterns across years (Liebgold et al., 2013), because our sample sizes were uneven across years, ranging from 19 to 71 individuals per year. Female sample sizes were especially low in some years (e.g., we had genetic data from only 6 individuals in 2004), and therefore, we analyzed all of the individuals together to improve our power to detect patterns of fine‐scale genetic structure and reduce the chances of error (Banks & Peakall, 2012). We ran the analysis for 999 permutations, following the protocol described by Peakall et al. (2003). We used a test of heterogeneity (Smouse, Peakall, & Gonzales, 2008) to determine whether spatial genetic structure existed within each sex and overall (i.e., both sexes combined). This analysis uses an omega test (ω) to determine whether the correlogram exhibits significant spatial structure against the null hypothesis of no spatial genetic structure. We also compared spatial genetic structure between sexes to determine whether males and females exhibited differences in spatial genetic structure. Similar to our overall analysis, we used Smouse and Peakall's test of heterogeneity to determine whether spatial genetic structure patterns are different between each sex against the null hypothesis of no difference in spatial genetic structure between sexes (Smouse et al., 2008). Tests of heterogeneity were considered significant only when *p* < .01 (Smouse et al., 2008). Lastly, we tested for heterogeneity between sexes within each distance class using the squared paired‐sample *t* test (*t* ^2^). This test allowed us to make direct comparisons within each distance class and determine whether relatedness was significantly different between sexes.

### 2.6 Spatial acoustic structure analysis

In addition to analyzing fine‐scale genetic structure, we also analyzed the spatial acoustic structure of males and females. For this analysis, we wanted to know whether males and females exhibit similar patterns of song sharing. While song sharing decreases as distance between breeding territories increases, both generally (Podos & Warren, 2007; Tracy & Baker, 1999) and in this species specifically (Mennill & Vehrencamp, 2005), we wanted to examine whether spatial patterns of song sharing are comparable to spatial genetic structure patterns. If there are dispersal differences between the sexes, do patterns of song sharing reflect this? We conducted this analysis in GenAlEx, using the “multiple populations” analysis with the same settings, and binned distance classes that we used in the genetic analysis. Similar to our genetic analysis, we tested for heterogeneity overall, and also between sexes, using the previously described tests. Several other studies have employed spatial autocorrelation techniques in GenAlEx to analyze ecological and acoustic data (Pavlova et al., 2013; Peakall et al., 2003), demonstrating the suitability of this technique. To generate a pairwise distance matrix for acoustic dissimilarity, we converted our sharing coefficient (*S*_j_) to a dissimilarity value by subtracting *S*_j_ from 1. Again, we created separate distance matrices for each sex, including all 237 color banded individuals that we recorded full repertoires from in this analysis (134 males and 103 females).

### 2.7 Natal dispersal and song‐sharing analyses

We analyzed the relationship between song sharing and natal dispersal distance. Using all of the individuals identified as natal dispersers, we calculated the song sharing coefficient between all father–son pairs, and all mother–daughter pairs. For this analysis, we ran a multivariate linear regression model. We combined males and females together and used song sharing as our response variable and straight‐line natal dispersal distance and sex as our fixed variables. We also analyzed sexes separately, but for this analysis, we examined the relationship between song sharing and natal dispersal distance using a Pearson's correlation coefficient. We used this approach because our sample sizes were relatively small when the two sexes were analyzed separately. For both analyses, we used the log‐transformed natal dispersal distance, as opposed to the raw distances, which violated assumptions of normality and variance. We excluded 2 males and 3 females because we did not have complete song repertoire data for the individual that dispersed. All statistical analyses were performed in SPSS (version 23.0; SPSS Inc., Chicago, IL, USA).

## 3 RESULTS

### 3.1 Dispersal

**Figure 2:**
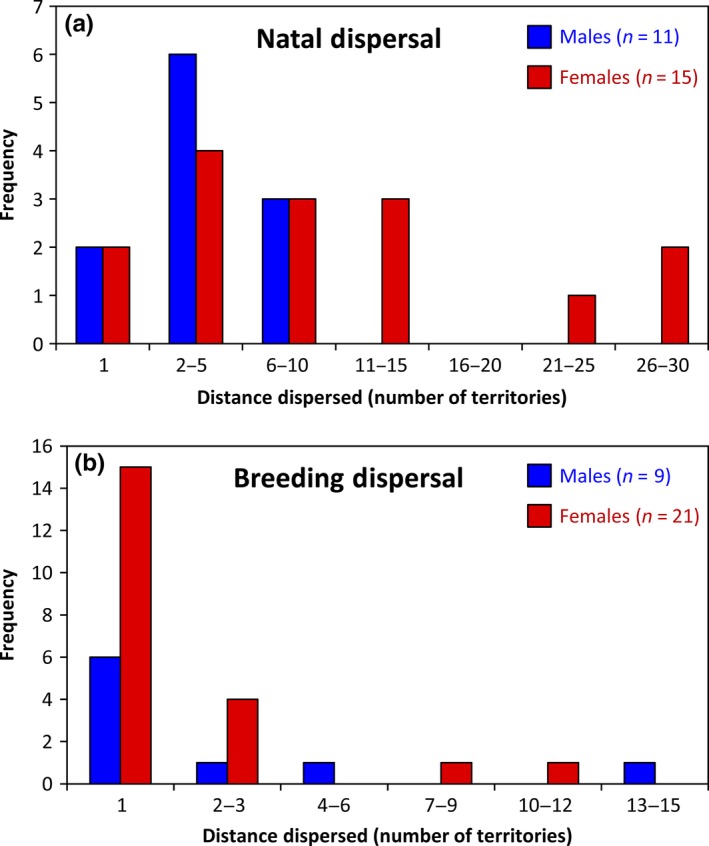
(a) Natal dispersal of Rufous‐and‐white Wrens measured as the number of territories dispersed before establishing their first breeding territories. Overall males dispersed fewer territories from their natal territories than females. (b) Female and male breeding dispersal, measured as the number of territories individuals dispersed before establishing a new breeding territory. Overall males and females dispersed relatively short distances, given that 70% of individuals moved into an adjacent breeding territory

Using both recapture/re‐sight data and genetic analysis to identify parent–offspring dyads, we identified 26 natal dispersal events by male (*n* = 11) and female (*n* = 15) Rufous‐and‐white Wrens (21 dispersal events identified through recapture/re‐sight data, and 18 dispersal events identified using microsatellite genotyping, 13 of which were also identified through capture/re‐sight data). Our combined analysis of both sexes revealed that males and females dispersed 1,234 ± 257 m between their natal territory and their first breeding territory. This distance was equivalent to a movement of 7.2 ± 1.5 territories from their natal territories. Between‐sex comparisons suggest that natal dispersal is female‐biased (Fig. 2a); dispersal from natal territories was significantly greater in females for both straight‐line distance (females, 1,644 ± 397 m, range = 121–4,561 m; males, 675 ± 190 m, range = 113–2,141 m; GLMM: −0.89 ± 0.44, *z *=* *2.04, *p* = .04) and the total number of territories an individual crossed (females, 9.7 ± 2.4 territories, range = 1–30; males, 3.8 ± 0.9 territories, range = 1–9; GLMM: −0.96 ± 0.35; *z *=* *−2.72, *p* = .01).

Over the 11 years of our study, we observed 30 breeding dispersal events, with females dispersing from one breeding territory to another more often than males (17 of 103 females and 7 of 134 males dispersed from one breeding territory to another; χ^2^ = 8.14, *p* = .005). Five individuals dispersed from breeding territories more than once: two females dispersed into a neighboring territory on two separate occasions, a third female dispersed from her breeding territory on three separate occasions, while two males dispersed into a neighboring breeding territory, but eventually returned to their original territory. Breeding dispersal distance estimates reveal that these movements were mostly local: Breeding males and females dispersed only 388 ± 83 m or 2.2 ± 0.5 territories (Fig. 2b). We observed a nonsignificant tendency for the difference between sexes in straight‐line breeding dispersal distance (females, 310 ± 73 m, range, 100–1,379 m; males, 572 ± 214 m, range, 100–2,200 m; GLMM: 0.61 ± 0.59, *z *=* *1.04, *p* = .30) and a nonsignificant difference for the number of territories that an individual dispersed across (females, 2.0 ± 0.5 territories, *n* = 21, range, 1–10 territories; males, 2.7 ± 1.2 territories, *n* = 9, range, 1–12 territories; GLMM: 0.35 ± 0.38, *z *=* *0.93, *p* = .35), although the lack of a statistical significance may be the result of limited statistical power due to the low number of recorded male breeding dispersal events.

Comparisons of natal dispersal patterns and breeding dispersal indicate that juvenile dispersal is likely to have a greater influence on genetic and acoustic structure. Comparisons of straight‐line distance indicate that individual's disperse greater distances from natal territories than breeding territories (GLMM: 1.15 ± 0.35, *z *=* *3.30, *p* = .001). Similar to straight‐line distance results, individuals dispersed more territories during natal dispersal events in comparison to breeding dispersal events (GLMM: 1.18 ± 0.26, *z *=* *4.54, *p* < .001).

### 3.2 Spatial genetic structure

**Figure 3:**
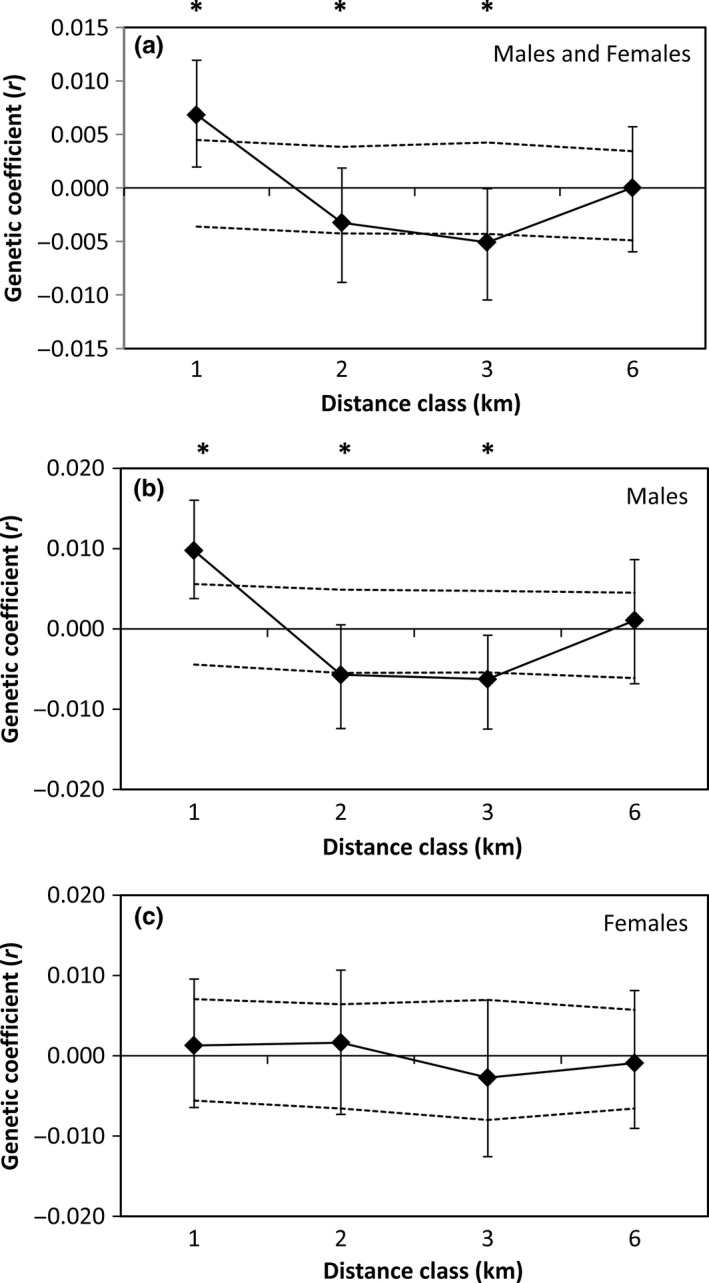
Correlograms showing the spatial genetic autocorrelation (*r*) with the designated distance classes for (a) males and females combined, (b) males only, and (c) females only. Male Rufous‐and‐white Wrens were more genetically similar at the closest distance class, but became more dissimilar at distances of 2 and 3 km. By comparison, females exhibited no significant genetic structure at any of the four distance classes. Dashed black lines represent the 95% upper and lower confidence limits determined using bootstrapping. Asterisks denote the distance classes where song sharing was significantly higher or lower from what was expected by chance (*p* < .05)

Rufous‐and‐white Wrens exhibited significant spatial genetic structure (ω = 31.81, *p* = .001; Fig. 3a; Table 1); individuals were more closely related to individuals at the closest distance class (1 km, *r *=* *.007, *p* = .001), but were less closely related to individuals at the two intermediate distance classes (2 km, *r *=* *−.006, *p* = .049; 3 km, *r *=* *−.006, *p* = .005). Males and females exhibited contrasting patterns of spatial genetic structure, and although these differences were not significant overall or between distance classes (ω = 3.96, *p* = .431; *t* ^2^ = 0.09–1.86, *p* > .17), our results indicate that dispersal is female‐biased and that males exhibit greater philopatry. While spatial genetic structure was significant for males (ω = 33.75, *p* = .002; Fig. 3b), female spatial genetic structure was not significant (ω = 9.81, *p* = .333; Fig. 3c). Female genetic structure was not significant at any of the four distance classes (*p* > .24). Males exhibited significant genetic structure at three of the four distance classes (1, 2, and 3 km); males were more closely related at the closest distance class (1 km, *r *=* *.01, *p* = .002), and were less closely related at the next two distance classes (2 km, *r *=* *−.006, *p* = .018; 3 km, *r *=* *−.006, *p* = .018).

**Table 1:** Results of genetic and acoustic spatial autocorrelation analyses of all birds combined and within each sex considered separately for Rufous‐an‐white Wrens. Measurement indicates the sex and level (genetic vs. acoustic) measured. *N* equals the number of pairwise comparisons for a given distance class for the calculation of *r*;* r* equals the correlation for each distance class; 95% CI represents the confidence intervals for each distance class, *p* denotes the significance of tests for *r *>* *0 if *r* is positive and *r *<* *0 if *r* is negative (α = .05); ω equals the test of heterogeneity value, *p*_ω_ denotes the significance of heterogeneity test (α = .01)

Measurement	1 km				2 km				3 km				6 km				ω	*p*_ω_
	*N*_1 km_	*r*_1 km_	95% CI_1 km_	*p*_1 km_	*N*_2 km_	*r*_2 km_	95% CI_2 km_	*p*_2 km_	*N*_3 km_	*r*_3 km_	95% CI_3 km_	*p*_3 km_	*N*_6 km_	*r*_6 km_	95% CI_6 km_	*p*_6 km_		
All birds genetic	**3,409**	**.007**	**0.004 to −0.004**	**.001**	**2,867**	**−.003**	**0.004 to −0.004**	**.049**	**2,736**	**−.005**	**0.004 to −0.004**	**.005**	2,496	.000	0.004 to **−**0.005	.504	**31.81**	**.001**
Males genetic	**2,217**	**.010**	**0.006 to −0.004**	**.002**	**1,881**	**−.006**	**0.005 to −0.005**	**.018**	**1,924**	**−.006**	**0.005 to −0.005**	**.012**	1,481	.001	0.005 to **−**0.006	.363	**33.75**	**.002**
Females genetic	1,192	.001	0.007 to **−**0.006	.338	986	.002	0.006 to **−**0.007	.322	812	**−**.003	0.007 to **−**0.008	.227	1,015	−.001	0.006 to **−**0.007	.367	9.31	.333
All birds acoustic	**4,141**	**.04**	**0.006 to −0.006**	**.001**	3,802	.001	0.005 to **−**0.006	.400	**3,437**	**−.024**	**0.005 to −0.007**	**.001**	**2,784**	**−.026**	**−0.006 to 0.006**	**.001**	**43.05**	**.001**
Males acoustic	**2,578**	**.058**	**0.009 to −0.007**	**.001**	2,388	.003	0.008 to **−**0.009	.201	**2,190**	**−.032**	**0.008 to −0.010**	**.001**	**1,755**	**−.050**	**0.008 to −0.009**	**.001**	**43.13**	**.001**
Females acoustic	**1,563**	**.013**	**0.008 to −0.007**	**.001**	1,414	**−**.003	0.007 to **−**0.007	.235	**1,247**	**−.015**	**0.007 to −0.008**	**.002**	1,029	.003	0.007 to **−**0.008	.289	**31.78**	**.001**

Bold values indicate tests that were significant at their respective p‐values.

### 3.3 Spatial acoustic structure

**Figure 4:**
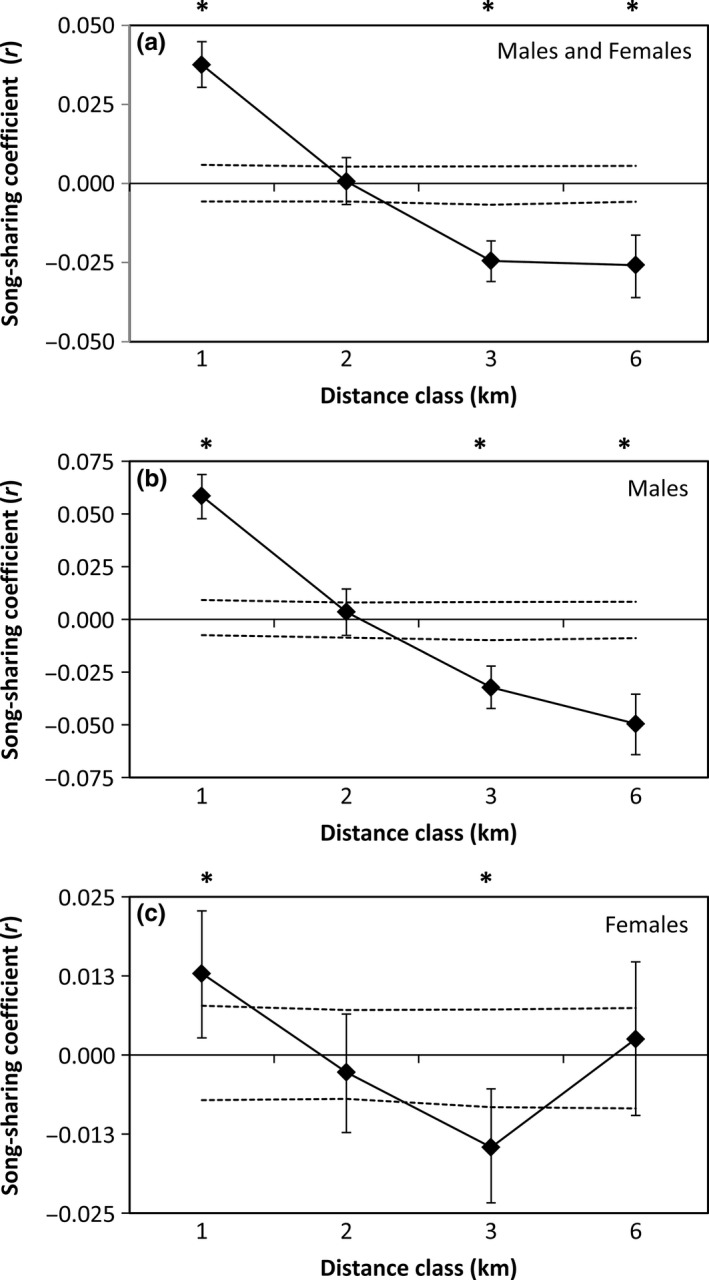
Correlograms showing the spatial acoustic autocorrelation (*r*) with the designated distance classes for (a) males and females combined, (b) males only, and (c) females only. Male and female Rufous‐and‐white Wrens had more similar repertoires at the closest distance class, but repertoires became more dissimilar as distance increased, although for females repertoire, sharing was not significantly different from random at the furthest distance class. Dashed black lines represent the 95% upper and lower confidence limits determined using bootstrapping. Asterisks denote the distance classes where song sharing was significantly higher or lower from what was expected by chance (α = .05)

Rufous‐and‐white Wrens exhibited significant spatial acoustic structure (ω = 43.28, *p* = .001; Fig. 4a; Table 1). Individuals shared more songs within the closest distance class (1 km, *r* = .038, *p* = .001) and shared fewer songs at the two farthest distance classes (3 km, *r *=* *−.024, *p* = .001; 6 km, *r* = −.026, *p* = .001; Fig. 4a). When the sexes were analyzed separately, males and females showed similar patterns of significant spatial acoustic structure (males, ω = 43.13, *p* = .001, Fig. 4b; females, ω = 31.78, *p* = .001, Fig. 4c), but spatial acoustic structure was significantly different between sexes (ω = 18.58, *p* = .001). Males exhibited greater song sharing than females at the closet distance class (1 km, males, *r *=* *.058, *p* = .001; females, *r *=* *.013, *p* = .001; *t* ^2^ = 28.99, *p* = .001), but shared fewer songs than females at the two furthest distance classes (3 km, males, *r *=* *−.032, *p* = .002; females, *r *=* *−.015, *p* = .002; *t* ^2^ = 5.46, *p* = .02; 6 km, *r *=* *−.050, *p* = .001; females, *r *=* *.003, *p* = .29; *t* ^2^ = 26.12, *p* = .001). Overall, spatial acoustic patterns suggest that males share more songs with neighbors (i.e., birds <1 km away) than do females, and that song sharing decreases with distance.

### 3.4 Song sharing and natal dispersal distance

**Figure 5:**
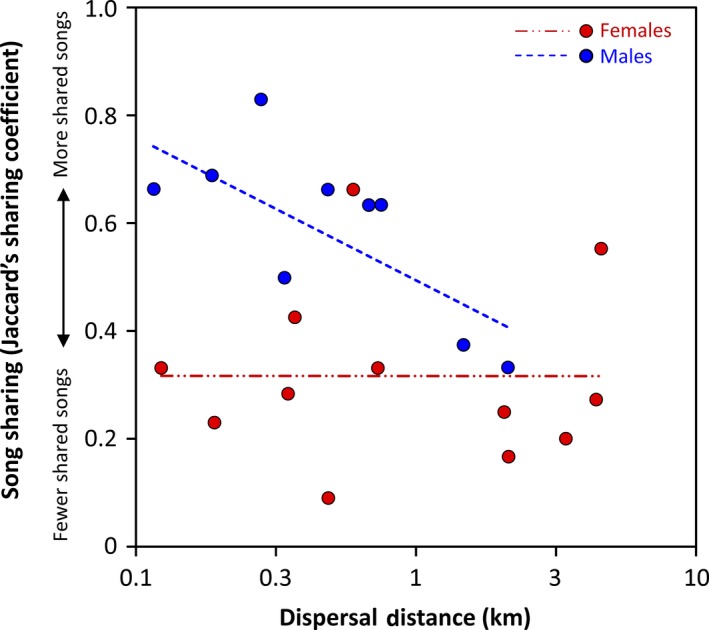
Song sharing (with the parent of the same sex) significantly decreases with natal dispersal distance in male Rufous‐and‐white Wrens (open circles) but not females (closed circles). For our analysis of males and females together, dispersal distance did not significantly predict song sharing (*t* = −1.13, *p* = .27). Dotted line shows the relationship for males (*r* = −.74, *p* = .02), while the dashed line shows the relationship for females (*r* = −.01, *p* = .99). The *x*‐axis shows values in km, on a log scale

Using adjusted Jaccard's coefficients of song sharing, we found that song sharing between sons and fathers was 0.59 ± 0.05, while song sharing between daughters and mothers was 0.32 ± 0.05. For our linear regression analysis of males and females combined, we found a statistically significant model (*F*_2,18_ = 8.16, adjusted *R* ^2^ = .42, *p* = .003), showing that sex was a significant predictor of song sharing with the parent of the same sex (parameter estimate: −0.25 ± 0.07, *t* = −3.46, *p* = .003), and not dispersal distance (parameter estimate: −0.09 ± 0.08, *t* = −1.13, *p* = .27). When we analyzed sexes separately, however, we found that males and females demonstrated contrasting relationships between song sharing and dispersal distance. Sons shared fewer songs with their fathers the farther they dispersed from their natal territory (*r* = −.74, *p* = .02, *n* = 9; Fig. 5), whereas the number of songs a daughter shared with her mother was not correlated with natal dispersal distance (*r* = −.01, *p* = .99, *n* = 12).

## 4 DISCUSSION

We combined field observation data and molecular genetic data to quantify dispersal distances and dispersal patterns in a long‐term study of Rufous‐and‐white Wrens. Our analysis of natal dispersal distance and spatial genetic structure indicate that dispersal is female‐biased in this tropical songbird. This result matches the widespread pattern of female‐biased dispersal common to many bird species (Clarke et al., 1997; Greenwood & Harvey, 1982). Furthermore, our results suggest that natal dispersal has a greater influence on spatial genetic structure and spatial acoustic structure than breeding dispersal. Overall, our results suggest that dispersal influences both genetic and acoustic structure in Rufous‐and‐white Wrens. Males exhibited more clustered genetic spatial structure and shared more songs with neighbors than non‐neighbors. By comparison, females exhibited no significant genetic spatial structure, and while females shared more songs with neighbors than non‐neighbors, the level of sharing was around four times lower than that observed in males. Current models attribute acoustic divergence to factors like intersexual and intrasexual selection in conjunction with dispersal (Ellers & Slabbekoorn, 2003; Wilkins et al., 2013), and our results emphasize that dispersal differences also influence acoustic variation.

### 4.1 Patterns of dispersal

Many tropical species occupy territories throughout the year (Greenberg & Gradwohl, 1986, 1997; Morton, Derrickson, & Stutchbury, 2000; Tobias, Gamarra‐Toledo, García‐Olaechea, Pulgarín, & Seddon, 2011), demonstrate high local recruitment (Gill & Stutchbury, 2006; Woltmann et al., 2012), and are thereby thought to exhibit limited dispersal (Moore, Robinson, Lovette, & Robinson, 2008; but see Van Houtan, Pimm, Halley, Bierregaard, & Lovejoy, 2007). Although sex‐biased dispersal has been more commonly studied in temperate species (Clarke et al., 1997; Greenwood & Harvey, 1982; Liebgold et al., 2013), our study adds to the body of work that has demonstrated sex‐biased dispersal in tropical species (Berg, Eadie, Langen, & Russell, 2009; Pavlova et al., 2013; Ribeiro, Lloyd, Feldheim, & Bowie, 2012; Sankamethawee et al., 2010; Vangestel, Callens, Vandomme, & Lens, 2013; Williams & Rabenold, 2005; Yáber & Rabenold, 2002). Our direct measurements of natal dispersal distances are comparable to those observed in several other tropical bird species, providing further insight into the movement of young animals living at tropical latitudes (e.g., Martin & Bucher, 1993; Woltmann et al., 2012; Woodworth, Faaborg, & Arendt, 1998). It is important to note that our estimates of dispersal are conservative, especially since our analysis is biased toward individuals that settled in our population; given that there is available habitat outside of our study area, some individuals may have dispersed farther and settled into territories outside of our study area. Although breeding dispersal is commonly observed in some species (Ribeiro et al., 2012), our estimates suggest that breeding dispersal is infrequent (only 10% of banded individuals switched breeding territories), female‐biased, and occurs over relatively short distances (Mulder, 1995; Woodworth et al., 1998; Yáber & Rabenold, 2002). These patterns indicate that natal dispersal has a greater influence than breeding dispersal on spatial acoustic structure and spatial genetic structure (Newton, 2007).

Similar to other nonmigratory bird species, in both the North Temperate Zone and the Tropics, we detected stronger spatial genetic structure for males than females in Rufous‐and‐white Wrens (Liebgold et al., 2013; Ribeiro et al., 2012; Sankamethawee et al., 2010; Vangestel et al., 2013; Yáber & Rabenold, 2002). Overall, our results show that tropical species may not be as sedentary as previously thought (Stutchbury & Morton, 2001). In particular, the dispersal capabilities of females reported here add to the growing literature suggesting that tropical birds may be capable of moving farther distances than we have recognized historically (Van Houtan et al., 2007). While our results indicate that males are more philopatric than females, it is noteworthy that dispersal patterns may vary among years. Whereas long‐term patterns may indicate female‐biased dispersal, dispersal patterns may show no bias or even male bias in some years (as in Eikenaar, Brouwer, Komdeur, & Richardson, 2010; Richardson, Ewen, Armstrong, & Hauber, 2010; Liebgold et al., 2013).

### 4.2 Spatial structure of songs

In Rufous‐and‐white Wrens, males and females showed similar spatial acoustic structure, sharing more songs with their nearest neighbors, but males exhibited stronger spatial acoustic structure than females (Mennill & Vehrencamp, 2005). Generally, studies of duetting species have shown that males exhibit higher song sharing and syllable sharing than females (Brown & Farabaugh, 1997; Hall, Rittenbach, & Vehrencamp, 2015; Mennill & Vehrencamp, 2005), although there are exceptions (e.g., Colombelli‐Négrel, 2016). Differences between sexes in our study suggest that between‐sex dispersal differences likely influence acoustic spatial structure. These results are unsurprising, given that dispersal has been shown to influence song diversity and spatial acoustic structure in other birds (Fayet, Tobias, Hintzen, & Seddon, 2014; Pavlova et al., 2013). Intersexual and intrasexual selections are also proposed drivers of acoustic divergence (Ellers & Slabbekoorn, 2003). While these factors may contribute to spatial acoustic patterns, it appears that dispersal is also an important factor in driving acoustic variation. In Rufous‐and‐white Wrens, dispersal is limited in males, and males exhibit greater neighbor–neighbor song sharing than females, as well as more spatial genetic structure. Females, by comparison, disperse greater distances and exhibit lower rates of neighbor–neighbor song sharing and no significant genetic structure.

The timing of song learning (predispersal vs. postdispersal) is expected to have a strong effect on whether dispersal influences pattern of geographic variation in vocal signals (Ellers & Slabbekoorn, 2003). Between‐sex differences in song sharing may also reflect sex‐specific tutor differences. Based on acoustic similarities, Evans and Kleindorfer (2016) found that male and female Superb Fairy‐wrens (*Malarus cyaneus*) learn song elements from both their social fathers and mothers. Studies of two temperate songbirds, in contrast, suggest that young males learn songs from natal neighbors and breeding territory neighbors (Nelson & Poesel, 2014; Wheelwright et al., 2008). In our study, we observed that sons share fewer songs with their fathers the farther they disperse from their natal territories. By comparison, the number of songs that daughters share with their mothers showed no relationship with natal dispersal distance. These results suggest that males learn songs postdispersal and primarily from breeding territorial neighbors (Payne, Thompson, Fiala, & Sweany, 1981; Wright, Rodriguez, & Fleischer, 2005). In contrast, female song‐learning patterns are less clear, although spatial patterns of acoustic structure suggest that repertoires are more similar between neighbors, consistent with the idea that similar patterns of postdispersal learning may apply to females. The lower rates of song sharing and the weaker patterns of spatial acoustic structure we observed for females may be a by‐product of dispersal differences between sexes. For example, males appear to move to the nearest available breeding territory and are thereby exposed to a limited number of potential song tutors (on average males dispersed only four territories away from their natal territories). In contrast, due to greater dispersal distances of females, young females may encounter more song tutors, either through their own movements, or by the movements of other females, thus resulting in lower levels of spatial acoustic structure. Alternatively, if dispersal is delayed in females (as is observed in some tropical species; Gill & Stutchbury, 2010; Russell, 2000; Russell, Yom‐Tov, & Geffen, 2004; Tarwater & Brawn, 2010), individuals may learn more songs from their mothers or natal territory neighbors, thereby explaining the nonsignificant relationship observed between natal dispersal and the proportion of songs shared between mothers and daughters.

Alternatively, between‐sex differences in song sharing may reflect differences in the way that male and female birds use their songs and repertoires. For example, male Bay Wrens (*Cantorchilus nigricapillus*) use their songs to communicate with both males and females: Male songs are used to attract females when males are unpaired, and acoustically guard mates from rival males when males are paired. By comparison, female Bay Wrens do not appear to use their songs to attract mates, but instead use their songs to defend territories against conspecific females (Levin, 1996a, 1996b). During territorial displays, male birds often match songs with neighbors (reviewed in King & MacGregor, 2016), and males often share a high proportion of songs or song types with their neighbors (Beecher, Campbell, Burt, Hill, & Nordby, 2000; Nelson, 2000; Trillo & Vehrencamp, 2005). Sharing songs with territorial neighbors may bestow several advantages, including increased reproductive success, and increased territory tenure (Beecher & Brenowitz, 2005; Beecher et al., 2000; Payne & Payne, 1997). Additionally, song sharing may reflect physiological condition and population of origin (Stewart & MacDougall‐Shackleton, 2008). Although song‐type matching is well known in males, there are fewer examples of it in females (see Marshall‐Ball, Mann, & Slater, 2006; Marshall‐Ball & Slater, 2004). Similar to male song, female song is a multifunctional signal, and even though some female birds use their songs to defend territories and mates (Cain & Langmore, 2015; Illes, 2014; Levin, 1996b; Logue, 2007; Templeton, Rivera‐Cáceres, Mann, & Slater, 2011; Tobias & Seddon, 2009), others use their songs primarily for communicating with their breeding partners (i.e., locating them in densely vegetated habitats) or coordinating breeding activities (i.e., nest building; Hall et al., 2015; Mays et al., 2006; Mennill & Vehrencamp, 2008; Templeton, Ríos‐chelén, Quirós‐guerrero, Mann, & Slater, 2013). In duetting species, repertoires may serve additional functions, including territory defense or mate guarding (Hall, 2004). Therefore, matching song types or phrases with mates may be more important than matching conspecifics in duetting species (Logue, 2007; Marshall‐Ball et al., 2004), especially since some duetting species adhere to duet codes (where males and females answer each other's songs with specific song types; Logue, 2006; Templeton, Mann, et al., 2013).

Across species where females sing, males and females not only vary in their vocal output, but also in how they use their songs. Differences in acoustic variation may reflect selection differences between sexes (Hall et al., 2015; Mennill & Rogers, 2006; Tobias et al., 2011) but they may also reflect developmental or song‐learning differences between sexes (Beecher & Brenowitz, 2005). For example, neuroanatomical studies have demonstrated that the song‐control regions of male songbirds are larger than the song‐control regions of female songbirds, and that differences in song output are related to the volume of the song‐control region (Macdougall‐Shackleton & Ball, 1999). Rufous‐and‐white Wrens also exhibit sexual dimorphism with respect to the volume of the song‐control region, and these differences correspond with repertoire size differences between sexes (Brenowitz & Arnold, 1986). Patterns of song ontogeny, and song‐learning patterns, remain poorly understood in female songbirds (Riebel et al., 2005), and therefore, further research is necessary to expand our knowledge of how these differences affect acoustic structure.

## 5 CONCLUSION

Like many other vertebrate species, Rufous‐and‐white Wrens display sex‐biased dispersal. Males settle near to their natal territories, whereas females disperse farther from their natal territories. Our results reveal a relationship between dispersal and acoustic variation in a tropical songbird where both sexes sing. We found a strong correlation between the level of song sharing between fathers and sons and dispersal distance, whereas we found no relationship between dispersal distance and the level of song sharing between mothers and daughters. These results indicate that males learn songs from territorial neighbors, and we suggest that this behavior may be important if song matching plays a role during social interactions between males. Females share fewer songs with neighbors than males do, suggesting that song matching is less important for females. Additionally, the lack of matching with neighbors could arise because females are learning songs throughout the dispersal process as they search for and assess potential breeding partners and breeding territories. Finally, natal dispersal, but not breeding dispersal, appears to shape the spatial acoustic structure of males and females, given that breeding dispersal is infrequent and occurs only over short distances. Taken together, our results provide insight into behavioral differences and cultural differences between male and female tropical birds.

## DATA ACCESSIBILITY

Data for this study have been archived in Dryad: https://doi.org/10.5061/dryad.3kn78.

## CONFLICT OF INTEREST

None declared.

## AUTHOR CONTRIBUTIONS

DJM initiated the long‐term study in 2003 and, together with his students, banded, recorded, and collected all blood samples; BAG leads the field investigation from 2011 to 2013, performed all laboratory work, and analyzed the genetic and acoustic datasets. BAG, DDH, and DJM designed the study and shared in writing the manuscript.
